# P93 - The role of allergy evaluation in children with eosinophilic esophagitis

**DOI:** 10.1186/2045-7022-4-S1-P148

**Published:** 2014-02-28

**Authors:** Maria Zande, Aspassia Angelakopoulou, Konstantinos Pitsios, Ekaterini Politi, Kleanthis Kleanthous, Ioanna Panagiotou, Eleftheria Roma-Giannikou, Ekaterini Syrigou

**Affiliations:** 1 General Hospital, Athens, Greece; 2 The Hospital for Sick Children, G.O.S, London, Greece; 3 Areteeio University Hospital, Cytopathology Department, Athens, Greece; 4 University Hospital, Athens, Greece; 5 Children's Hospital Agia Sofia, 1st Pediatric Clinic University of Athens, Athens, Greece

## Aim

Eosinophilic esophagitis (EoE) is a chronic inflammatory disease of the esophagus immune/antigens mediated. It is clinically characterised by symptoms related to esophageal dysfunction and associated with eosinophil-predominant esophageal inflammation. The role of atopy has been clearly demonstrated both in epidemiological and experimental studies and has important implications for diagnosis and therapy.The aim of this study was to assess the relationship between food allergy and eosinophilic esophagitis in the pediatric population, as well as the effect of dietary modifications in patients’ clinical symptoms.

## Methods

Thirty-six children aged 7 months to 12 years (median age 80 months), with EoE (esophageal symptoms, biopsy with >15 eosinophils/HPF after the patients have been treated with a PPI for at least 4–8 weeks and other causes have been excluded) were included to group I. Twenty age and sex matched, apparently healthy, infants and children were studied as control group(group II).Serum specific IgEs to cow milk, egg, wheat, rice, corn, soy, chicken, potato, beef, peanut and pork were measured with the CAP-FEIA. Skin prick tests and atopy patch tests (using fresh foods) were performed for the same allergens.

## Results

All children in control group had negative CAP, SPT and APT to all food allergens. In group I 30/36 children (83%) had positive APT. Of these, 12/30 (40%) had also positive SPT and 16/30 (53%) had also positive CAP. Of the 30 positive children 4 (4/30, 13%) had positive APT to one food allergen, 3/30 (10%) had positive APT to two food allergens and 23/30 (77%) to 3 or more food allergens. In the APT-positive children, in group I, withdrawing the suspected food allergens for an 8 week period resulted in the improvement of symptoms.

## Conclusion

Food allergens seem to be a significant etiologic factor for eosinophilic esophagitis in infants and young children. Skin tests are able to identify, in most of the cases, the responsible food allergens leading to dietary modifications and symptom remission.

